# Impairment of NKG2D-Mediated Tumor Immunity by TGF-β

**DOI:** 10.3389/fimmu.2019.02689

**Published:** 2019-11-15

**Authors:** Mariya Lazarova, Alexander Steinle

**Affiliations:** Institute for Molecular Medicine, Goethe-University Frankfurt am Main, Frankfurt am Main, Germany

**Keywords:** NK cell, NKG2D, TGF-β1, tumor immune evasion, immunotherapy

## Abstract

Transforming growth factor-β (TGF-β) suppresses innate and adaptive immune responses via multiple mechanisms. TGF-β also importantly contributes to the formation of an immunosuppressive tumor microenvironment thereby promoting tumor growth. Amongst others, TGF-β impairs tumor recognition by cytotoxic lymphocytes via NKG2D. NKG2D is a homodimeric C-type lectin-like receptor expressed on virtually all human NK cells and cytotoxic T cells, and stimulates their effector functions upon engagement by NKG2D ligands (NKG2DL). While NKG2DL are mostly absent from healthy cells, their expression is induced by cellular stress and malignant transformation, and, accordingly, frequently detected on various tumor cells. Hence, the NKG2D axis is thought to play a decisive role in cancer immunosurveillance and, obviously, often is compromised in clinically apparent tumors. There is mounting evidence that TGF-β, produced by tumor cells and immune cells in the tumor microenvironment, plays a key role in blunting the NKG2D-mediated tumor surveillance. Here, we review the current knowledge on the impairment of NKG2D-mediated cancer immunity through TGF-β and discuss therapeutic approaches aiming at counteracting this major immune escape pathway. By reducing tumor-associated expression of NKG2DL and blinding cytotoxic lymphocytes through down-regulation of NKG2D, TGF-β is acting upon both sides of the NKG2D axis severely compromising NKG2D-mediated tumor rejection. Consequently, novel therapies targeting the TGF-β pathway are expected to reinvigorate NKG2D-mediated tumor elimination and thereby to improve the survival of cancer patients.

## Introduction

Transforming growth factor-β (TGF-β) is a potent suppressor of immune responses affecting many subsets of immune cells in various ways (1). For example, TGF-β impairs MHC class II expression (2, 3), thus potentially impairing priming of CD4 T cells, and suppresses the activity of cytotoxic lymphocytes by inhibiting the differentiation, proliferation, and effector functions of CD8 T cells and NK cells (1, 4). TGF-β also promotes the differentiation of suppressive immune cells subsets (5–7). In physiological settings, the TGF-β-mediated immune suppression is crucial for the establishment of immune tolerance and prevention of chronic inflammation, e.g., in the gastrointestinal tract (4, 8, 9), but in malignant disease TGF-β promotes immune escape, tumor progression and metastasis (4, 10–13). Importantly, there is emerging evidence that TGF-β also impairs immunorecognition of tumor cells by NK cells and cytotoxic T cells through down-regulation of activating immunoreceptors such as NKG2D. NKG2D ligation by stress-induced MHC class I-like glycoproteins on tumor cells transmits a potent stimulatory signal into cytotoxic lymphocytes and therefore promotes immunosurveillance of malignant cells (14, 15). Hence, evasion from NKG2D-mediated recognition is thought to represent a major mechanism allowing tumors to escape from tumor immunity. In this review, we specifically focus on the TGF-β-mediated impairment of immunorecognition through the NKG2D axis and its implications for tumor immunity and cancer therapies. The function and biology of TGF-β as well as of NKG2D will be summarized only briefly as both have extensively been reviewed elsewhere (4, 10, 16–18).

## TGF-β: Expression, Receptors, and Signaling

The three members of the human TGF-β family, TGF-β1,−2, and−3 are synthesized as precursor proteins containing an N-terminal latency-associated peptide (LAP) (~280–300 amino acids) followed by a shorter C-terminal polypeptide (112–114 amino acids) which represents the biologically active mature cytokine (19, 20). During the intracellular processing of this precursor protein, LAP is cleaved but remains associated with the TGF-β dimer forming an inactive latency complex that is sequestered into the extracellular matrix. Activation of TGF-β requires release from this latency complex (21). In addition, TGF-β can be present on the surface of regulatory T cells (Tregs), endothelial cells, platelets, macrophages and microglia in a membrane-associated form (22, 23). Mature TGF-β homodimers bind, with or without the assistance of the accessory receptor betaglycan (BG, also called TGF-β receptor III), first to homodimers of the TGF-β receptor II (TGF-βRII) which then phosphorylate TGF-β receptor I homodimers (TGF-βRI, ALK5) under formation of a hexameric complex of TGF-β, TGF-βRII, and TGF-βRI homodimers. Subsequently, TGF-βRI phosphorylates the cytoplasmic SMAD2 and SMAD3 proteins, which then, under association with SMAD4, transmigrate into the nucleus and exert transcriptional activity (4, 16). TGF-β receptors are expressed on virtually all immune cells. Of note, TGF-βRII expression was shown to decline in the course of mouse NK cell maturation (24).

## TGF-β-Mediated Immunosuppression

TGF-β1 is the predominant TGF-β family member expressed by immune cells and suppresses innate and adaptive immune responses at multiple levels (4, 25). Amongst others, TGF-β has a prominent role in dampening T and NK cell responses: TGF-β impairs T cell proliferation and effector functions through down-regulation of IL-2 during T cell priming (26) and has been shown to induce cell cycle arrest and apoptosis of T cells (27–29). TGF-β directly inhibits the cytotoxic functions of CD8 T cells (30) and the differentiation of both Th1 and Th2 subsets by downregulation of their key transcription factors (31–35). Further, TGF-β downregulates the expression of MHC class II molecules via affecting CIITA expression (2, 3) thus impairing the capacity of antigen presenting cells (APC) for antigen presentation and CD4 T cell priming. TGF-β also inhibits the expansion, cytotoxicity, and cytokine production by NK cells (36–39). More recently, TGF-β was shown to block the IL-15-induced metabolic activity and proliferation of NK cells by inhibiting mTOR activity (24). In addition, TGF-β promotes conversion of NK cells into non-cytotoxic ILC1 in the tumor microenvironment (TME) thereby blunting tumor killing (40). TGF-β further promotes differentiation of Tregs (5, 6) and of myeloid derived suppressor cells (MDSC) (7). An eminent importance of TGF-β in affecting cancer immunosurveillance and efficacy of checkpoint blockade cancer therapy was recently highlighted by a series of studies on human cancer patients and of mouse tumor models: TGF-β produced by the TME was shown to restrict tumor infiltration by T cells and other cytotoxic lymphocytes and to block the acquisition of a Th1 effector phenotype (41–43). Inhibition of TGF-β activity not only facilitated T cell infiltration into central sites of the tumor, but also unleashed vigorous and efficient anti-tumor immunity, particularly in the course of checkpoint blockade (41–43). On the other hand, immunosuppression by TGF-β plays a central physiologic role in the establishment of immune tolerance and control of inflammation. Germline deletion of TGF-β1 in mice is lethal due to multi-organ inflammation (8, 9). Loss of TGF-β signaling, particularly in T cells, is associated with uncontrolled adaptive T cell responses and severe inflammatory disease (4, 44–46). In the persistent presence of antigen stimuli, e.g., in the gastrointestinal tract, TGF-β aids in suppression of immune responses in order to prevent chronic inflammation (4).

## Pleotropic Role of TGF-β in the Development and Progression of Tumors

Loss of function mutations in the TGF-β receptors or in SMAD proteins are found in many tumors indicating a function as a tumor suppressor (4). TGF-β inhibits cell growth (4, 47–50), blocks the transition of pre-malignant cells to a more evasive phenotype and induces their apoptosis (51, 52). In contrast, there is also broad evidence suggesting that TGF-β supports tumorigenesis and invasiveness, and enables tumor growth by establishing an immunosuppressive and T cell excluding TME. For example, elevated TGF-β levels in the TME impair anti-tumor T cell responses (11–13, 53) with restricting T cell infiltration into the tumors as shown for mouse models of metastatic colorectal, urothelial and epithelial ovarian cancers (41–43). TGF-β is thought to function as a tumor suppressor at the early stages of tumor development, but with the progression of disease, cancer cells may decouple growth-inhibitory paracrine TGF-β signals by obstructing their TGF-β receptor signaling pathway and rather exploit the immunosilencing capacity of TGF-β to facilitate immune evasion and metastatic dissemination (4, 16).

## NKG2D-NKG2DL Axis

NKG2D is a type II transmembrane glycoprotein comprising an extracellular C-type lectin-like domain, a transmembrane domain, and a short cytoplasmic portion without signaling motifs (54, 55). NKG2D glycoproteins form disulfide-linked homodimers with both monomers building a single ligand binding site (56). In humans, NKG2D homodimers associate with two pairs of DAP10 adaptor proteins through interaction of charged residues in the respective transmembrane domains. Formation of this hexameric complex is required for cell surface expression of NKG2D and signal transduction (55, 57). NKG2D is found on virtually all human NK cells and CD8 T cells, on most γδ T cells and iNKT cells, as well as on a few CD4 T cells (54, 58). Ligation of NKG2D activates cytotoxicity and cytokine production of NK cells and provides stimulatory signals for effector CD8 T cells (54, 59–63). NKG2D expression is enhanced through cytokines promoting NK and T cell survival and expansion such as IL-15 and IL-2 (62–64).

NKG2D ligands (NKG2DL) are stress-inducible membrane-bound proteins distantly related to MHC class I molecules. In human, there are two families of NKG2DL, the MIC family consisting of MICA and MICB, and the ULBP family consisting of ULBP1 through ULBP6 (14, 65, 66). All NKG2DL contain an ectodomain with an MHC class I-like α1α2-fold (14, 56, 67), but unlike MHC molecules NKG2DLs neither associate with β2-microglobulin, nor present antigenic peptides (54, 61). MICs contain an additional Ig-like α3 domain in their extracellular part that is absent from ULBPs (14, 56). Most MICs are single-pass transmembrane proteins, although there are also reports for GPI-anchored MICA variants (68, 69). ULBP1 through ULBP3 and ULBP6 are GPI-anchored, whereas ULBP4 and ULBP5 are inserted into the membrane with a single transmembrane domain (67, 70, 71).

While NKG2DLs are typically absent from the cell surface of healthy cells, NKG2DL transcripts are found in almost all human tissues (72), indicating a dominant control of NKG2DL expression at the post-transcriptional level. NKG2DL are surfacing on activated hematopoietic cells which may contribute to an NKG2D-mediated regulation of immune responses and may dampen T cell responses (73, 74), e.g., during the resolution of an infection (75, 76). NKG2DL are also found on many human tumor cell lines and primary human tumors (77), and are up-regulated during viral infections, particularly during infections with viruses of the herpesvirus family (78, 79). Such NKG2DL expression marks infected or malignant cells as “dangerous” for the immune system and facilitates their clearance through cytotoxic lymphocytes. NKG2DL on tumor cells enhance their susceptibility to NK cell killing (54, 80), protects against tumor initiation (81) promotes tumor rejection and/or reduce the tumor progression (82–85). Tumors utilize a variety of mechanisms to escape from NKG2D-mediated immunosurveillance: these mechanisms include the release of soluble NKG2DLs (sNKG2DL) either by proteolytic cleavage (71, 86–88) or by exosomal release of membrane-bound NKG2DLs (89, 90). Release of sNKG2DL reduces the density of NKG2DL on malignant cells and thereby impairs NKG2D-mediated recognition and elimination of tumor cells by cytotoxic lymphocytes (82–85). While some studies also report down-modulation of surface NKG2D on cytotoxic lymphocytes through sNKG2DL-mediated internalization (91, 92), other studies were unable to confirm these findings or attributed NKG2D down-modulation instead to TGF-β (82, 93, 94). Possibly, potent NKG2D down-modulation by TGF-β (see below) in serum samples of cancer patients containing both TGF-β and sNKG2DL may have led to some erroneous conclusions regarding sNKG2DL-mediated NKG2D down-modulation in previous studies (15, 92–94). Also, sera of tumor-free MICA-transgenic mice containing very high levels of sMICA did not affect NKG2D surface levels by splenic mouse NK cells (82). However, persistent exposure of NKG2D to membrane-bound MICA down-regulated surface NKG2D and reduced NK cell cytotoxicity in these MICA-transgenic mice as well as in other transgenic mouse models overexpressing NKG2DL (82, 95, 96). Hence, strong overexpression of NKG2DL may represent a strategy of tumor cells to blunt NKG2D-mediated immunosurveillance. In contrast to proteolytically shed monomeric sNKG2DL (i.e., most MICA variants, MICB, and ULBP2), exosomally released NKG2DL such as the prevalent MICA^*^08, ULBP1 or ULBP3 may down-modulate surface NKG2D through multivalency-based cross-linking (89, 90). Further escape mechanisms from NKG2D-mediated cancer immunosurveillance include down-regulation of NKG2DL through miRNAs (97, 98), epigenetic changes or transcriptional repression (99, 100), and TGF-β mediated signaling as outlined below. Intraindividual heterogeneity of malignant cells can also impair NKG2D-mediated tumor clearance: a recent study by Paczulla et al. showed that malignant cells of human acute myeloid leukemia (AML) patients are heterogeneous for NKG2DL expression with leukemic stem cells (LSC) being devoid of NKG2DL and therefore resistant to NK cell-mediated elimination (100). Poly-ADP-ribose polymerase 1 (PARP1) was shown to repress transcription of NKG2DL in LSC thereby enabling their escape from NKG2D-mediated immunosurveillance (100).

## TGF-β Impairs NK and T Cells Function Through Interference With the NKG2D Axis

**Figure 1:**
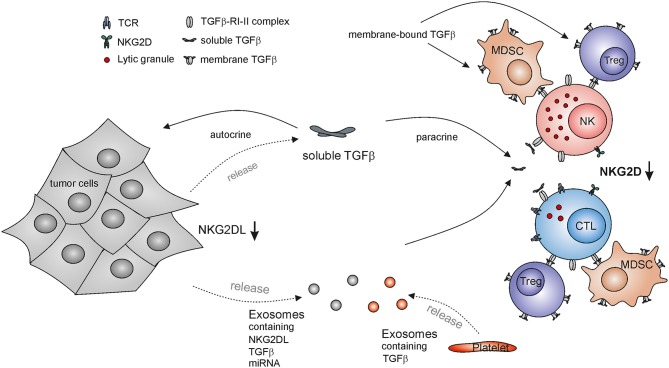
TGF-β-mediated escape from NKG2D-mediated tumor immunorecognition by cytotoxic lymphocytes. NKG2D down-regulation on cytotoxic lymphocytes impairs their immunosurveillance of NKG2DL-expressing malignant cells and subsequent tumor elimination. Tumor cells release both soluble TGF-β and TGF-β-containing exosomes locally and systemically acting on NK cells and cytotoxic T lymphocytes (CTL), thereby inducing downregulation of NKG2D. In addition, tumor-derived exosomes may contain NKG2DLs and miRNA with the capacity to down-regulate NKG2D surface expression. TGF-β also acts on tumor cells in an autocrine or paracrine manner thereby reducing NKG2DL expression and further subverting cancer immunosurveillance by the NKG2D-NKG2DL axis. Other major source of TGF-β are platelets as well as regulatory T cells (Tregs) and myeloid derived suppressor cells (MDSCs) which also present membrane bound TGF-β.

Soon after cloning of the TGF-β1 cDNA (101), TGF-β1 was shown to inhibit both the proliferation of T cells (102) and the anti-tumor cytotoxicity of NK cells (36). While it was demonstrated that TGF-β impairs effector functions of NK cells against target cells, the underlying mechanisms remained elusive until it was reported by Moretta and colleagues that TGF-β downregulates the surface expression of the activating NK receptors NKG2D and NKp30, thereby impairing NK cytolysis of tumor cell lines *in vitro* (103) (Figure 1). Obviously, this effect depends on the extent of expression of NKG2DL and ligands of NKp30 by the respective tumor cells. Subsequent studies confirmed and extended these observations (104, 105): TGF-β inhibits NKG2D-mediated lysis of target cells without altering the expression of perforin or Fas ligand, or without affecting NK cell viability, indicating that down-regulation of NKG2D is a major effect of TGF-β on NK cytolysis of tumor cells (105). A study on glioblastoma not only reported TGF-β-induced reduction of NKG2D expression on NK cells, but also on cytotoxic T lymphocytes (CTL). Decreased NKG2D expression resulted in the decreased cytolysis of NKG2DL positive targets by NK cells and a reduced NKG2D-mediated co-stimulation of CD8 T cells (104). The elevated TGF-β levels in sera of patients with lung and colorectal cancers were shown to down-regulate NKG2D on NK cells. Other studies linked increased tumor-associated TGF-β levels with the impairment of the function of NK cells and CTLs, and NKG2D down-regulation in various malignancies including Hodgkin lymphoma (106), gastric cancer (107) and head and neck squamous cell carcinoma (108, 109). Hence, impaired NKG2D expression may serve as a biomarker for TGF-β-compromised cytotoxic lymphocytes. TGF-β-mediated down-regulation of NKG2D and associated impaired NK cell functions were also reported in the context of infections with hepatitis B and C viruses (110, 111).

Elevated TGF-β levels as detected in glioblastoma patients were also shown to affect the expression of NKG2DLs (104, 112): experimentally reduced TGF-β expression by glioma cells led to an increase of MICA, ULBP2, and ULBP4 transcripts and increased cell surface expression of MICA and ULBP2 as well as of a reduction of tumorigenicity *in vivo* (104, 112). Thus, tumor derived TGF-β can act in a paracrine fashion to decrease NKG2D expression on cytotoxic lymphocytes in the TME and in an autocrine manner to diminish tumor-associated NKG2DL expression thereby impairing the innate recognition and clearance of tumors (104). Hence, TGF-β-mediated repression of NKG2DL expression together with proteolytic shedding of NKG2DL has been suggested to facilitate the immune escape of glioma in the immune-privileged brain (112). However, there are also some reports that TGF-β treatment increases surface levels of NKG2DLs (113). The induction of cell surface expression of MICA and MICB upon culture with TGF-β was described for several human cell lines and appears at least partially dependent on mTOR signaling. In the case of HaCat cells, the increase in NKG2DL was associated with the TGF-β-induced epithelial-to-mesenchymal transition (113). These reports indicate that the regulation of NKG2DL expression by TGF-β may be dependent on the cell type and the context of the microenvironment.

## Role of Membrane-Bound and Exosomally Secreted TGF-β

TGF-β can be presented as a membrane bound form on the surface of several cell types (22, 23) and there is evidence that membrane-bound TGF-β can also regulate NKG2D expression. Surface-bound TGF-β presented by Tregs was found to decrease NKG2D expression on NK cells and this correlated with the inhibition of NK cell cytotoxicity (114). Adoptive transfer of Tregs in Treg-deficient mice resulted in a decreased NKG2D expression and NK cell cytotoxicity *in vivo* and reduced the anti-tumor effector functions of NK cells in an NKG2D-sensitive tumor model in a TGF-β dependent manner (114). Other reports confirmed that TGF-β produced by Tregs impairs NKG2D-mediated NK cell killing of target cells *in vitro* (115). Decreased NKG2D expression was also found on NK cells in murine models of liver and lung cancer and correlated with the frequency of MDSC. MDSC isolated from cancer-bearing mice were able to impair NK cells functions and NKG2D expression on NK cells *in vitro*, and after adoptive transfer in healthy mice, and depletion of MDSC from tumor-bearing mice restored the functionality and NKG2D expression on NK cells and delayed the tumor progression *in vivo* (116). The observed effects were also mediated through a membrane-bound TGF-β presented by MDSC, while NK cells deficient in TGF-β signaling were resistant to the MDSC-mediated effects (116). Exosomal secretion of NKG2DL can impair NKG2D expression on cytotoxic lymphocytes thus desensitizing them for NKG2DL-mediated tumor recognition (89). Exosomes derived from a panel of tumor cell lines and from patients with malignant pleural mesothelioma were also shown to contain TGF-β on exosomes and down-regulated NKG2D on the surface of CTLs and NK cells. Neutralizing TGF-β or MICA of exosomes indicated that TGF-β, and not MICA, is the main factor driving the observed NKG2D downregulation (94). Microvesicles derived from sera of AML patients were also shown to contain high levels of TGF-β and decreased NKG2D expression as well as NK cell cytotoxicity in a TGF-β dependent manner (117).

## TGF-β in the Platelet-NK Cell Cross-Talk

Mouse models suggest that metastasis formation is dependent on the tumor-protective function of platelets, but the cross-talk between tumor-coating platelets and NK cells in the blood is not yet fully understood (118, 119). Platelet-derived TGF-β may promote the immune escape of circulating disseminated tumor cells as activated platelets release factors reducing the activation and IFN-γ production of NK cells and the expression of a set of activating NK cell receptors including NKG2D. This effect is at least partially mediated by platelet-derived TGF-β (120). Platelet-derived TGF-β was shown to induce an invasive phenotype of tumor cells promoting metastasis in mouse models of colon and breast carcinoma. Abrogation of either TGF-β signaling in tumor cells or TGF-β expression by platelets suppressed metastasis formation and epithelial-mesenchymal transition (121). Accordingly, it was proposed that platelet-derived TGF-β in the circulation provides a “pulse” to tumor cells enabling them to acquire a more invasive mesenchymal-like phenotype (121). Platelets were also shown to secrete TGF-β-rich exosomes upon storage, e.g., before transfusions, that induce downregulation of NKG2D, NKp30, and DNAM-1 and modulate NK cell functions (122).

## Mechanisms of TGF-β-Mediated Down-Regulation of NKG2D and NKG2DL

**Figure 2:**
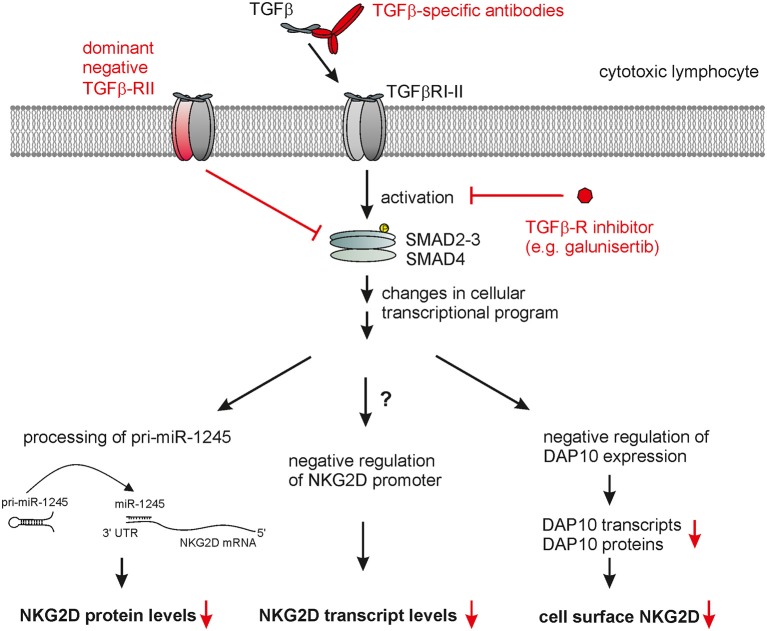
Therapeutic targeting of TGF-β-mediated NKG2D down-regulation by cytotoxic lymphocytes. TGF-β bound to a tetrameric complex of TGF-β-RI and TGF-β-RII homodimers causes phosphorylation of SMAD proteins, which, together with further contextual transcriptional regulators, alter the cellular transcriptional profile. This ultimately also leads to markedly reduced cell surface NKG2D expression by cytotoxic lymphocytes which appears to result from several direct and indirect effects: (i) decrease of NKG2D transcripts, (ii) maturation of miR-1245 interacting with the 3'-UTR of NKG2D transcripts thereby repressing NKG2D expression, and (iii) decreased levels of DAP10 transcripts and proteins with DAP10 being essentially required for NKG2D surface expression. Therapeutic strategies interfering with TGF-β signaling (marked in red) to rescue NKG2D expression include: (i) neutralization of TGF-β receptor through TGF-β specific antibodies or soluble TGF-β-RII, (ii) inhibition of TGF-β-RI-II activation through small molecules such as galunisertib, and (iii) engineering therapeutic lymphocytes prior to adoptive transfer with dominant negative TGF-β-RII chains.

The molecular mechanism underlying the TGF-β-mediated down-modulation of NKG2D surface expression are not yet fully elucidated. Several studies reported that TGF-β treatment only results in a moderate reduction of NKG2D transcripts (64, 103) demonstrating that TGF-β mainly acts through post-transcriptional mechanisms on NKG2D expression. A more recent study provided conclusive evidence that induction of mature miR-1245 by TGF-β controls NKG2D expression in NK cells (123) (Figure 2). TGF-β augments processing of the pri-miR-1245 in NK cells and strongly increases the levels of mature miR-1245 in NK cells which acts on a target site in the 3'-UTR of NKG2D transcripts. Overexpression or silencing of miRNA-1245 markedly reduced or enhanced surface NKG2D on NK cells, respectively (123). Of note, IL-15 suppressed the maturation of miRNA-1245 which is detectable in tumor-derived exosomes in hematopoietic malignancies (123). Expression of miRNA-1245 is up-regulated by c-myc which directly binds to the miRNA-1245 promoter (124) indicating that exosomes of c-myc-driven tumors may harbor miRNA-1245 and thereby target NKG2D expression. However, TGF-β-mediated reduction of surface NKG2D levels is not completely abolished in miR-1245 knock-out cells arguing for further mechanisms (123). Accordingly, other studies reported that TGF-β treatment substantially decreases DAP10 expression both at mRNA and protein levels (64, 125). Since NKG2D cell surface expression strictly depends on complex formation with DAP10 (55, 57), the TGF-β-mediated down-regulation of DAP10 indirectly complements the direct suppression of NKG2D expression by miR-1245 (64, 123).

Multiple miRNA have also been shown to down-regulate expression of human NKG2DL by human tumor cells thereby impairing NKG2D-mediated tumor recognition (97, 98, 126, 127). However, for most of these miRNA their tumor-associated regulation is not clear. In contrast, expression of the oncomiR-183, up-regulated by TGF-β in lung cancer, was shown to down-regulate MICA and MICB glycoprotein expression in lung tumor cell lines through a binding site in the 3'-UTR of MICA/B transcripts. Accordingly, shRNA-mediated knock-down of either TGF-β or miR-183 resulted in an enhanced MICA/B expression and cytolysis by CD8 T cells (128). TGF-β-induced miR-183 was also reported to impair expression and function of several activating NK receptors such as NKp44 through down-regulation of the adaptor protein DAP12 (129), and hence, targets tumor recognition by NK cells at various receptors.

## Rescue of the NKG2D-NKG2DL Axis in Cancer by TGF-β Targeting Therapies

The crucial role of TGF-β in tumor progression and tumor immune escape renders this cytokine an important target for therapeutic intervention in cancer. Accordingly, multiple cancer therapies targeting the TGF-β pathway are currently being evaluated in clinical trials. Therapies targeting the TGF-β pathway have, amongst others, the potential to boost tumor elimination by cytotoxic lymphocytes through harnessing the NKG2D-mediated tumor recognition and boosting cytolysis by NK cells and cytotoxic T lymphocytes. For example, galunisertib (LY2157299), a small molecule inhibiting TGF-βRI kinase activity (Figure 2), prevented *in vitro* the TGF-β-mediated down-regulation of surface NKG2D (as well as of NKp30, DNAM-1, TRAIL) by activated NK cells and preserved their cytotoxic activity toward various tumor cell lines (130, 131). Accordingly, administration of galunisertib markedly enhanced the anti-tumor effect of adoptively transferred activated human NK cells in NSG mice bearing human tumors (130, 131). Significant therapeutic effects in phase II clinical trials were reported with galunisertib given either in combination with gemcitabine in pancreatic cancer (132) or as a monotherapy in hepatocellular carcinoma (133). Importantly, no adverse side effects and no cardiac toxicity were reported by several clinical trials (134). Encouraging pre-clinical studies show that a combined cancer treatment using galunisertib together with checkpoint blockade antibodies strongly potentiated cancer immunity (43, 135).

Suppressive effects of TGF-β may also be overcome by targeted delivery of cytokines IL-2, IL-15, and IL-18 into the tumor. While TGF-β was shown to have a dominant effect over IL-2 or IL-15 alone with regard to NKG2D modulation on the surface of NK cells (64, 105), a combination of IL-2 and IL-18 protected NK-92MI cells from TGF-β-mediated NKG2D down-regulation and the associated impairment of NK cell function (136). An IL-15 superagonist/IL-15Rα fusion complex (ALT-803) rescued NK cytolysis of tumor cell lines from TGF-β1-mediated immunosuppression *in vitro* and diminished TGF-β1-mediated down-regulation of surface NKG2D (137). IRX-2, a poorly defined mixture of cytokines derived from the culture supernatants of activated lymphocytes, was tested in clinical trials for treatment of head and neck squamous cell cancer, and increased NKG2D surface expression and NKG2D-dependent NK cytotoxicity, even in the presence of TGF-β (109).

TGF-β-neutralizing macromolecules such as TGF-β-specific mAb or soluble forms of TGF-βRII are currently evaluated in several phase I and II clinical trials in treatment of patients with various solid tumors (4). A recent report on a phase I/II clinical trial for treatment of chemo-refractory metastatic breast cancer with the TGF-β-neutralizing mAb fresolimumab during radiotherapy did not observe an objective or abscopal response in tumor patients treated with fresolimumab (138, 139). Exploratory analyses of circulating T cells from these patients indicated that this treatment regimen with fresolimumab was not sufficient to reverse the impaired T cell function observed in these cancer patients (139). In addition, various chimeric molecules consisting of soluble TGF-βRII receptors, acting as TGF-β traps, linked to checkpoint blockade antibodies currently are tested in pre-clinical studies and clinical trials. Several preclinical studies have already shown substantially enhanced anti-tumor responses as compared to a monotherapy with anti-CTLA4 or anti-PD-L1 mAb in various mouse solid tumor models (140, 141). For example, administration of a bifunctional fusion protein, termed M7824, with an anti-PD-L1 mAb coupled to the extracellular domain of TGFβ-RII, provided an efficient tumor control in preclinical models of colorectal and breast tumors. M7824 administration resulted in a shift of tumor-infiltrating immune cell populations toward an increase of cytotoxic CD8 T cells and NKG2D^+^NKp46^+^NK cells which mediated tumor immunity (141). M7824 has already given to a small cohort of heavily pretreated patients with advanced solid tumors showing early signs of efficacy and a manageable safety profile (142), and is currently undergoing further clinical trials in patients with advanced solid tumors (e.g., NCT02517398, NCT02699515).

An elegant approach to shield adoptively transferred cytotoxic lymphocytes from the suppressive effects of TGF-β in cancer immunotherapy, such as NKG2D silencing, is the transduction of T cells or NK cells with a dominant negative form of TGF-βRII prior to adoptive transfer (143, 144). Transduction of cord blood NK cells with a dominant negative TGF-βRII efficiently blocked TGF-β signal transduction and supported the maintenance of the cell surface expression of activating receptors and NK cell cytotoxicity in the presence of TGF-β (144). Treatment of a small cohort of chemorefractory Hodgkin lymphoma patients with TGF-βRII-transduced autologous EBV-derived tumor antigen-specific CD8 T cells showed complete remission in four out of seven patients (145) suggesting that this type of engineered cytotoxic lymphocytes is safe and efficacious.

Another elegant strategy attempts to convert immunosuppressive signals of soluble TGF-β into stimulatory signals using the chimeric antigen receptor (CAR) concept. A recent report created a chimeric receptor consisting of a TGF-β-binding scFv fused to the transmembrane segment of CD28 and the cytoplasmic signaling domains of both CD28 and CD3ζ (146). T cells ectopically expressing such a CAR were activated by TGF-β-induced CAR dimerization that led an activation of both NFAT and NFκB with a subsequent stimulation of Th1 cytokine responses and an enhanced T cell expansion (146). It will be of great interest to address the *in vivo* performance of such anti-TGF-β CAR T cells utilizing TGF-β as an activating growth factor in mouse models of solid tumors.

## Concluding Remarks

TGF-β broadly and potently suppresses the effector functions of NK cells and cytotoxic T lymphocytes with the TGF-β-mediated impairment of the NKG2D axis representing an important facet of this phenomenon in cancer immunity. Down-regulation of both NKG2D, on cytotoxic lymphocytes, and NKG2DL surface expression, on tumor cells, facilitates the immune escape of tumor cells from induced-self recognition and elimination by cytotoxic lymphocytes. Hence, targeting TGF-β appears to represent a key intervention for an efficient boosting of tumor immunity and should be considered in future cancer treatment modalities. However, the intracellular mechanisms mediating the suppression of the NKG2D axis through TGF-β are not yet fully elucidated and further research is needed to define the underlying molecular and cellular pathways to allow for the development of more tailored and efficacious therapeutic options.

## Author Contributions

ML and AS wrote the manuscript.

### Conflict of Interest

The authors declare that the research was conducted in the absence of any commercial or financial relationships that could be construed as a potential conflict of interest.
